# Graphene Quantum Dots and Enzyme-Coupled Biosensor for Highly Sensitive Determination of Hydrogen Peroxide and Glucose

**DOI:** 10.3390/ijms19061696

**Published:** 2018-06-07

**Authors:** Bingdi Wang, Jing Shen, Yanjun Huang, Zhenning Liu, Hong Zhuang

**Affiliations:** 1 College of Food Science and Engineering, Jilin University, Changchun, Jilin 130062, China; wangbd16@mails.jlu.edu.cn (B.W.); shenjing9915@163.com (J.S.); huangyanj@jlu.edu.cn (Y.H.); 2 Key Laboratory of Bionic Engineering (Ministry of Education), College of Biological and Agricultural Engineering, Jilin University, Changchun, Jilin 130022, China; liu_zhenning@jlu.edu.cn

**Keywords:** graphene quantum dots, bio-enzyme detection, glucose, enzyme-coupled biosensor, quenching method

## Abstract

In this paper, a simple and specific graphene quantum dots (GQDs)-based fluorescent biosensor adopted for the determination of glucose based on the combination of the enzyme-coupled method and fluorescence quenching mechanism is demonstrated. Glucose was oxidized by the enzyme glucose oxidase (GOx), forming hydrogen peroxide (H${}_{2}$O${}_{2}$) via the catalysis by horseradish peroxidase (HRP). H${}_{2}$O${}_{2}$ was then employed to oxidize phenol to quinone, which led to effective quenching effect in the GQDs–GOx–HRP–phenol system. By optimizing the reaction conditions of the GQDs-enzyme system, a linear relationship between the concentration of glucose and the fluorescence intensity over a range of 0.2–10 μmol/L was obtained. The limit of detection for glucose is 0.08 μmol/L. The present biosensor for the determination of glucose showed satisfactory reproducibility and accuracy in human serum samples. Since the enzymes have high specificity and unique affinity to the certain substance, the enzyme-coupled system promises a sensitive way for further detection of those chemicals which could be oxidized by enzymes and generated H${}_{2}$O${}_{2}$ or glucose. GQDs and other fluorescent materials coupled with several enzymes can be applied to extensive sensing field.

## 1. Introduction

Glucose is a basic and widely-used monosaccharide that can be found easily in both human bodies and other species. Glucose acts as the raw material and the intermediate product of respiration and metabolism, and an unbalanced glucose level in the human body can lead to such disease as diabetes which is considered a principal health problem that negatively affects millions of people in the world [1,2]. Glucose monitoring and diabetes diagnostic have been continuous concerns among the public. The fast and accurate monitoring of glucose level, mainly in blood, is necessary for diagnostic and treatment [3,4]. In recent years, various kinds of methods have been applied in glucose quantitative analysis in different samples, including electrometric [5], voltammetric [6], colorimetric [7] and fluorimetric detection [8,9]. Fluorescence detection is a current research interest in analyzing food [10], diagnosing of diabetes [11,12], etc. Because of the convenient implementation and high sensitivity, several types of bio-probes have been made using enzymes and some fluorescent material for fast and deep sensing detection [13,14].

Enzymes and coenzymes have been extensively used in sensing system owing to their high activity and specificity, acting as biosensors in the electrochemical and optical area [15,16]. They are capable of changing function and structure, thus frequently modified onto the sensor or added in the solution to accelerate or change the process [17,18]. Compared with the electrochemical methods, optical methods are more reliable in low concentration analyte detection using the UV and fluorescence spectra. For instance, glucose oxidase, lactate enzyme, and galactose oxidase have been extensively exploited [15,19]. Glucose oxidase (GOx), because of its stability and sensitivity, is practicable in glucose detecting. GOx, based on a well-founded mechanism, is broadly used. During the process, glucose is oxidized by O${}_{2}$ with GOx, generating gluconolactone and H${}_{2}$O${}_{2}$ as oxidation products [20]. Horseradish peroxidase (HRP) is a kind of practicable enzyme in detecting H${}_{2}$O${}_{2}$ in the presence of hydrogen donor; the reaction between HRP and H${}_{2}$O${}_{2}$ is rapid and specific [21]. In recent years, due to the targeting function, those reactions based on enzymes received a great deal of attention.

Graphene quantum dots (GQDs) and its derivatives have been an area of intense investigation [22,23,24,25,26]. Graphene has been detected and used for years due to its excellent proprieties. When the size of quantum is <100 nm, the GQDs showed superior properties due to its specific nanometer size effect and specific shape [27,28]. Compared with common carbon dots [29], GQDs has drawn more and more attention these years, owing to its extraordinary properties and functions, including low toxicity [30], high fluorescence activity [31], high solubility [32], unique biocompatibility [33], and long-term resistance to photo-bleaching as well as the feasibility of functionalization at their edges [22,34]. Therefore, many experimental works, including Pb${}^{2+}$ [35], Cu${}^{2+}$ [36], Hg${}^{2+}$ [37], Mn${}^{2+}$ [38], Ag${}^{+}$ [39], Ce${}^{4+}$ [40], DHB [41], glucose [42], and telomere DNA [43] detection are based on its ultrahigh sensitivity and unique electronic and chemical properties.

This study applied a novel Graphene quantum dots (GQDs)-based fluorescent bio-probe for the analysis of glucose value, combining the enzymatic reaction and the quenching effect of GQDs. H${}_{2}$O${}_{2}$ formed in the glucose oxidation reaction catalyzed by GOx, and upon the addition of HRP and phenol, the phenol oxidized as caused by H${}_{2}$O${}_{2}$ and HRP [44] to quinone compounds, which could efficiently quench the fluorescence intensity of GQDs [41]. The quenching fluorescence intensity is proportional to the concentration of glucose. Compared with other works, the enzyme-coupled biosensor is much more selective and sensitive for the determination of glucose due to specific characteristics of enzymes. The determination of glucose is based on the combined effect of GOx and HRP, eliminating the common interference factors. The proposed method can pave the way for sensitive detection of analytes that can generate glucose and H${}_{2}$O${}_{2}$ such as lactate, cholesterol, glycogen, ascorbic acid (AA) and uric acid (UA). By combining GQDs or other fluorescent materials with enzymes, bi-enzymes or multi-enzymes biosensors can be synthesized and utilized for particularly effective analysis.

## 2. Results and Discussion

### 2.1. GQDs Characterization

**Figure 1:**
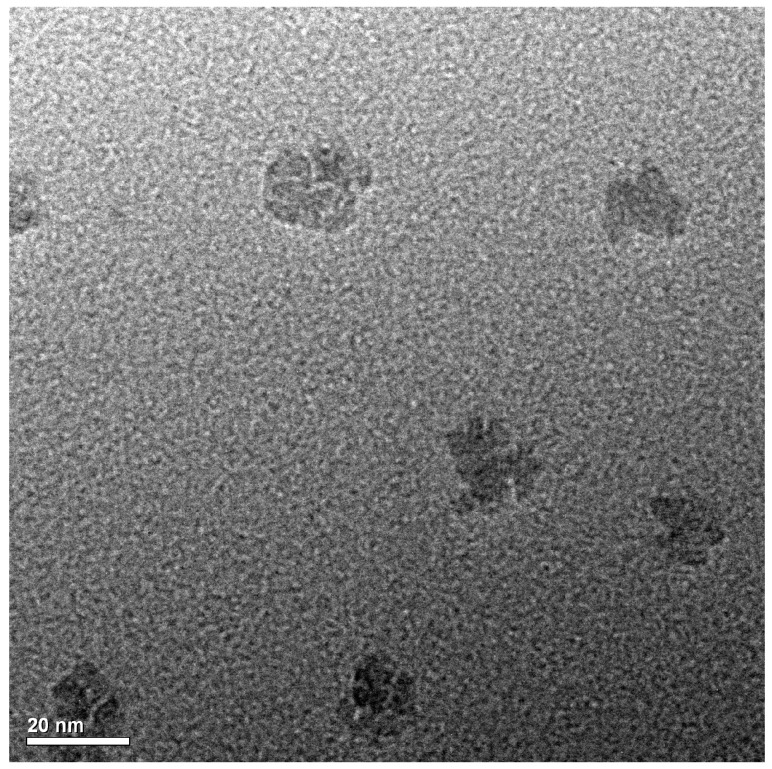
The transmission electron microscopy (TEM) image of GQDs.

After the preparation of GQDs, the prepared GQDs were characterized by transmission electron microscopy (TEM). As shown in Figure 1, the diameters of the GQDs are mainly distributed in the range of 10–20 nm. The above-observed properties were consistent with a previous report [25].

### 2.2. Detection Principle of the Enzyme-Coupled Biosensor

**Scheme 1:**
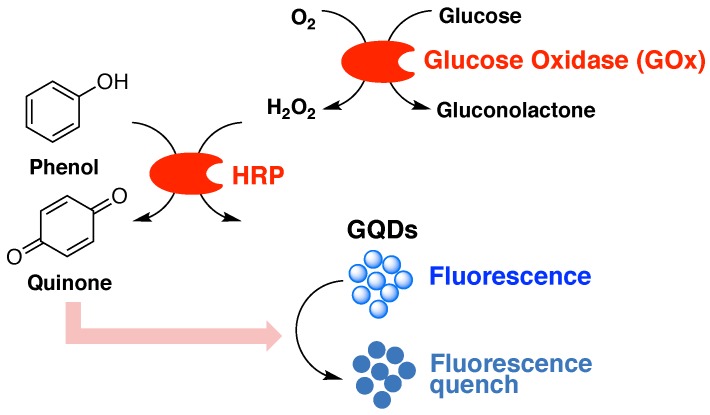
Schematic representation of sensing strategy to glucose by using glucose oxidase (GOx), horseradish peroxidase (HRP), phenol and GQDs catalyzed reaction.

**Figure 2:**
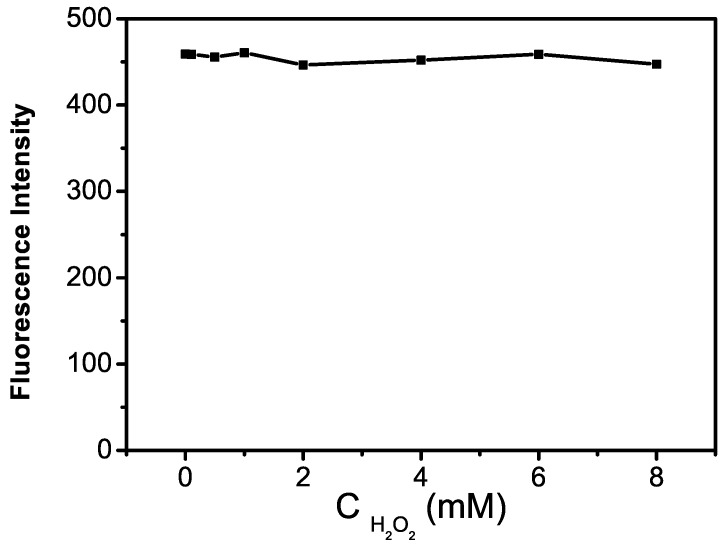
The effect of concentration levels of H${}_{2}$O${}_{2}$ on the PL intensity of GQDs.

**Figure 3:**
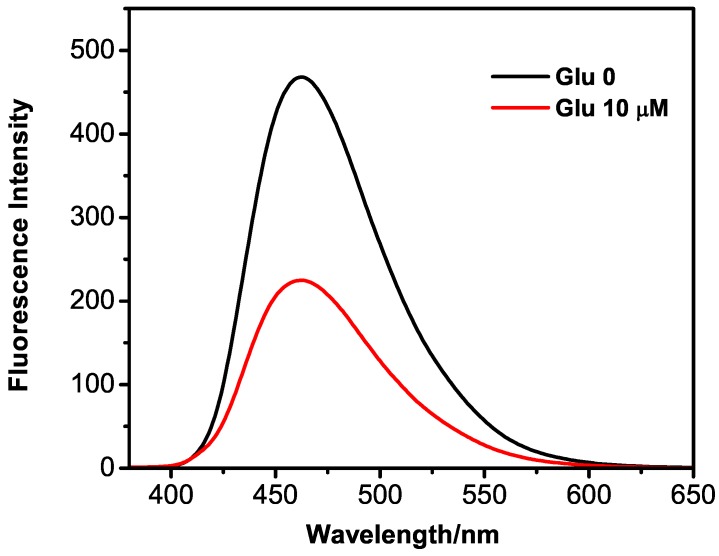
The effect of components of glucose and biosensor on the PL intensity of GQDs: (Black) GQDs–GOx–HRP–phenol; and (Red) GQDs–GOx–HRP–phenol with glucose addition.

The principle of GQD-glucose detection is based on the combination of glucose enzyme reaction and the quenching effect of quinone produced on the GQDs, and the idea of the sensing system is illustrated in Scheme 1. Glucose could cause the quenching effect of GQDs in GOx–HRP–phenol system. Several experiments based on control variate methods have been carried out, the fluorescence emissions were investigated, and the results are shown below. In Figure 2, in the presence of different concentrations of H${}_{2}$O${}_{2}$, the fluorescence emission of GQDs did not show apparent changes, suggesting that there was no interaction between GQDs and H${}_{2}$O${}_{2}$. According to a well-founded reaction mentioned above, glucose is oxidized by O${}_{2}$ and GOx and produced H${}_{2}$O${}_{2}$ and gluconolactone [45]. Thus, the influence of mere addition of glucose and GOx in the GQDs sensing system was very slight. Nevertheless, as shown in Figure 3, when glucose, GOx, HRP, phenol were added simultaneously to the GQDs solution, a significant decrease in fluorescence intensity could be observed. For the selectivity of GOx, the level of H${}_{2}$O${}_{2}$ is hinged on the concentration of glucose. In contrast to the first experiment, upon addition of phenol and HRP, phenol acted as hydrogen donor, which could rapidly and specifically be oxidized by H${}_{2}$O${}_{2}$ via catalysis by HRP, and generated quinone [18,46]. The primary oxidized product, quinone, was a good electron acceptor and efficiently transferred the electron from the excited GQDs [47], causing a considerable quenching effect of the fluorescent system [48]. The spectral results mentioned above provide evidence that, under the same condition, with the presence of GOx, HRP and phenol, glucose can be oxidized; the quinone generated is a suitable quencher in the assay; and the glucose concentration can effectively influence the fluorescence quenching degree of the system.

### 2.3. Optimization of the GQDs–GOx–HRP–Phenol Sensing System

**Figure 4:**
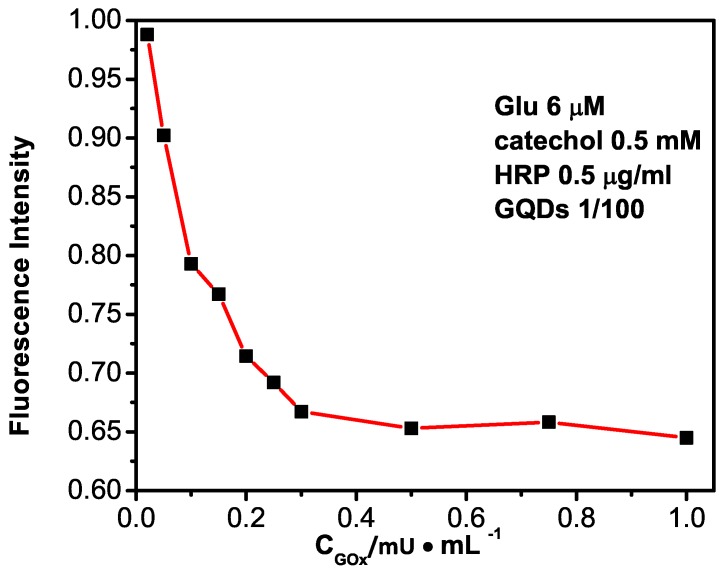
Effect of enzyme concentration on fluorescence intensity.

As the H${}_{2}$O${}_{2}$ concentration is related to the different addition of the glucose oxidase, to further increase the sensitivity of the system, different concentrations of the glucose oxidase (GOx) were added to the system that includes glucose, GQDs, phenol, and HRP under the same conditions, and the results were investigated by measuring the fluorescence spectra immediately. In Figure 4, it is clear that the quenching effect enhanced with the increasing concentration of GOx until the addition achieved 0.5 mM. The fluorescence intensity attained the minimum value at 1.0 mM. However, considering the high enzyme cost, 0.5 mM glucose oxidase was a suitable concentration for the following experiments.

**Figure 5:**
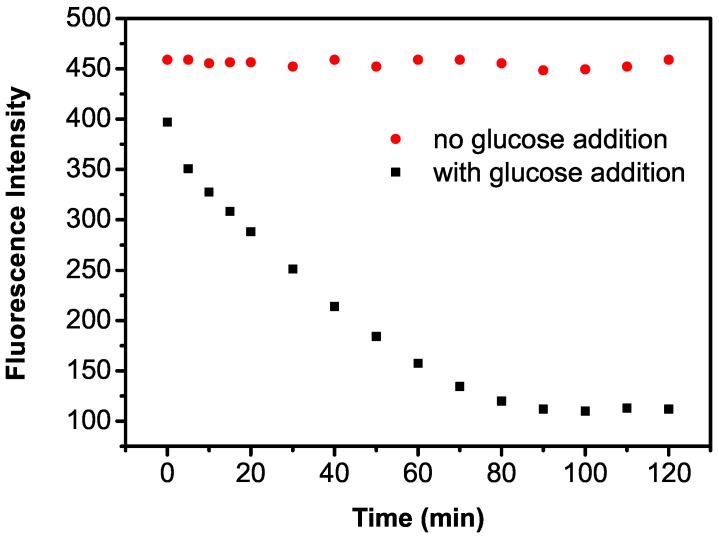
The relationship between fluorescence intensity of the GQDs and the incubation time in the: presence of glucose (Black); and absence of glucose (Red).

To detect the response time of the fluorescence intensity of catalyzed oxidation and GQDs to glucose, the time-dependent fluorescence changes of the system were observed, as shown in Figure 5. The red curve is with the absence of glucose, and the black curve shows the system including glucose. It is obvious that, upon the addition of glucose, fluorescence intensity fell considerably as time increased. After incubation time was longer than 80 min, the fluorescence intensity reached plateau and remained nearly unchanged. The reaction was completed within 80 min. In this study, the incubation time of 80 min was used to establish the optimal condition.

### 2.4. Detection of Glucose via the GQDs–GOx–HRP–Phenol Sensing System

**Figure 6:**
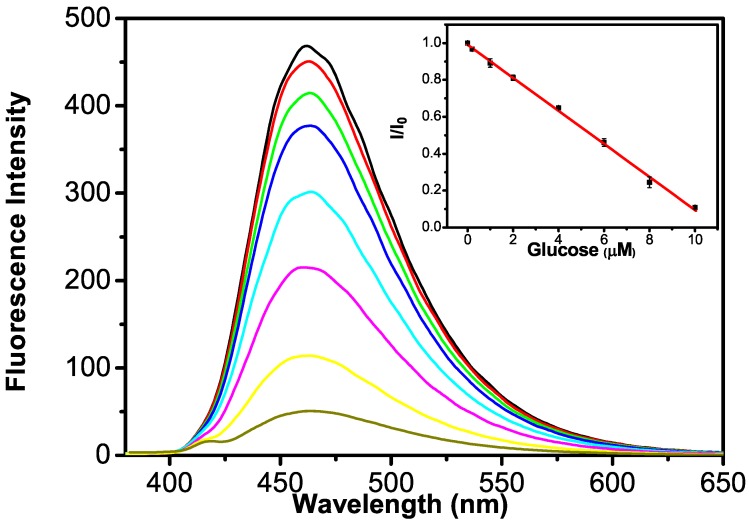
Fluorescence emission spectra of different concentrations of glucose. 0 (black), 0.2 (red), 1.0 (green), 2.0 (blue), 4.0 (cyan), 6.0 (magenta), 9.0 (yellow), 10 (dark yellow) μmol/L, respectively. Inset is the linear calibration of the fluorescence intensity ratio of I/I${}_{0}$ and the concentration of glucose.

To evaluate the sensitivity of this biosensor system to glucose, the experiments proceeded under the optimum conditions discussed above. The fluorescence intensity of GQDs–GOx–HRP–phenol system dropped proportionally upon increasing the concentration of glucose, and the maximum wavelength of the system kept constant (see Figure 6). Adding more glucose generated more quencher through the double enzyme reaction and enhanced the quenching effect on GQDs. As shown in the inset of Figure 6, glucose concentration showed a good linear correlation to I/I${}_{0}$ in the range of 0.2–10 μM (R${}^{2}$ = 0.999) (I and I${}_{0}$ were the fluorescence intensities of the GQDs–HRP–phenol system in the presence and absence of glucose, respectively). The regression equation for this method can be described as I/I${}_{0}$ = 0.991 − 0.0896C${}_{glucose}$ (μM) and the corresponding detection limit (LOD) was 0.08 μM, which was calculated as 3σ/s. The LOD is much lower than most previous reported colorimetric, electrochemical and fluorescent sensors for glucose detection (Table 1).

### 2.5. Selectivity of the GQDs–GOx–HRP–Phenol Sensing System for Detecting Glucose

**Figure 7:**
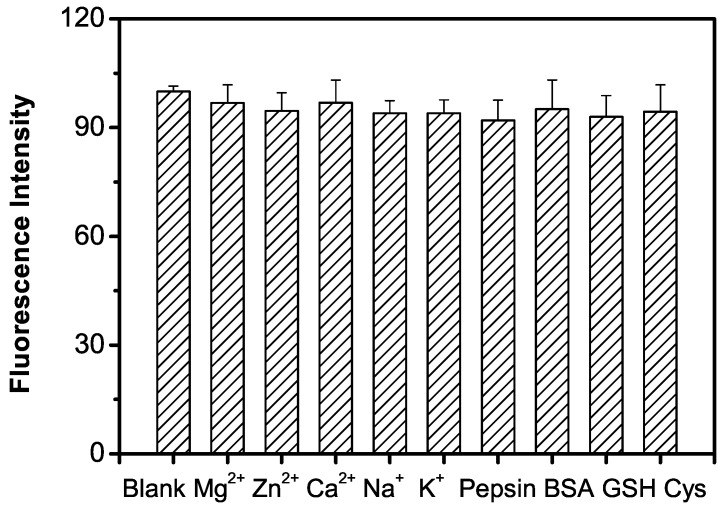
Effect of glucose concentration on I/I${}_{0}$ in the presence of potential interferents.

To test whether this GQDs–GOx–HRP–phenol sensing system is selective toward glucose, the fluorescence changes were measured upon the addition of eight potential substances that commonly exist in the human body,: Mg${}^{2+}$, Zn${}^{2+}$, Ca${}^{2+}$, Na${}^{+}$, K${}^{+}$, pepsin, BSA, GSH, and Cys. As the results in Figure 7 show, the fluorescence intensity observed of the GQDs–GOx–HRP–phenol sensing system did not change obviously over the high concentration of interference, which suggests that the present approach has excellent selectivity in detecting glucose with various kinds of substances. The high selectivity can be attributed to the specificity between enzyme and substrate: GOx is selective toward glucose and HRP is selective toward H${}_{2}$O${}_{2}$, while other interferents cannot bind the enzyme and the biosensor has strong anti-interference ability.

### 2.6. Application of Assaying Glucose Concentration in Human Serum Samples

To evaluate the applicability of the GQDs–GOx–HRP–phenol sensing system in the biological environment, we measured the fluorescence intensities of human serum samples spiked with glucose, and calculated the glucose concentration, recoveries and relative standard deviations (RSDs) for every system. The measurements of two samples were repeated three times and the results obtained from three separate experiments showed good repeatability, as listed in Table 2. The samples spiked with different concentrations showed good recoveries in the range of 98.5–102.0%, RSDs were less than ±5%, and the results were consistent with those obtained by medical methods. Therefore, we found that the enzyme-coupled biosensor is reliable and comparable in the determination of glucose in the biological environment.

## 3. Materials and Methods

### 3.1. Chemicals and Reagents

In this work, all reagents used were of analytical grade without further purification. Ultrapure water used throughout all experiments was purchased from Hangzhou Wahaha Group Co. Ltd., (Hangzhou, China). Catechol, NaOH, Na${}_{2}$HPO${}_{4}$, NaH${}_{2}$PO${}_{4}$, citric acid (CA) and H${}_{2}$O${}_{2}$ were acquired from Beijing Chemical Works (Beijing, China). Horseradish peroxidase (HRP), Pepsin, Bovine serum albumin (BSA), cysteine (Cys), glutathione (GSH), Ascorbic acid, Glutathione and amino acids were obtained from Beijing Dingguo Biotechnology (Beijing, China). Glucose and glucose oxidase (Worthington, Shanghai, China) were purchased from Sangon Biotech (Shanghai, China). Human serum samples were obtained from the healthy volunteers at the China Japan Union Hospital (Changchun, China) and the samples were prepared and diluted.

### 3.2. Instrumentation

Transmission electron microscopy (TEM) images were obtained with a JEM2100F high-resolution transmission electron microscope (JEOL, Tokyo, Japan) at an acceleration voltage of 200 kV. All measurements of fluorescence were performed under a 360 nm excitation wavelength. All samples were decanted in a standard 1 cm pathway spectrophotometric cuvette. The fluorescence signal corresponded the maximum emission of GQDs well at 466 nm. Records were obtained on a Shimadzu RF-5301 spectrometer (Shimadzu, Kyoto, Japan). Optical measurements were done in aqueous solutions at room temperature.

### 3.3. Synthesis of GQDs

The GQDs was synthesized in accordance with a method described in previous reports [41,42]. Briefly, 2.0 g citric acid is heated in a 5 mL beaker at approximately 260 ${}^{\circ }$C for 17 min to produce molten CA. The light yellow liquid was then introduced into 100 mL of 250 mM NaOH solution with vigorous stirring, neutralizing at a pH of 7.0. The obtained GQDs stored at 4 ${}^{\circ }$C for further application.

### 3.4. Optimization of the Sensing System

For H${}_{2}$O${}_{2}$ detection, H${}_{2}$O${}_{2}$ with different concentrations were freshly prepared and added with 0.5 μg/mL HRP, 0.05 mM catechol and 10% GQDs followed. All samples were incubated at 37 ${}^{\circ }$C for 80 min.

For glucose concentration effect analysis, 10 μmol/L glucose was added into the system with 5 μmol/L GQDs, 0.5 μg/mL HRP, 0.05 mM catechol, and 0.05 mM GOx. All samples were incubated at 37 ${}^{\circ }$C for 80 min.

For enzyme concentration effect analysis, fluorescence intensities were detected in the system of 6 μM glucose, 0.05 mM catechol, 0.5 μg /mL HRP and 1% GQDs. All samples were incubated at 37 ${}^{\circ }$C for 80 min.

For incubation time detection, glucose was added to the system within 5 μmol/L GQDs, 0.5 μg/mL HRP, 0.05 mM catechol, 10 μmol/L glucose and 0.05 mM GOx, and samples were incubated at 37 ${}^{\circ }$C for different periods.

All ingredients were blended well under the optimal condition before the fluorescence signal of the mixture was recorded with a luminescence spectrometer.

### 3.5. Assay of Glucose

The calibration curve of the approach toward glucose was carried out under optimal conditions. Glucose solutions with certain concentrations (0, 0.2, 1.0, 2.0, 4.0, 6.0, 9.0, and 10 μmol/L, respectively) were added into the system with 0.5 μg/mL HRP, 0.05 mM catechol, 0.05 mM GOx and 5.0 μmol/L GQDs, respectively. All samples were then diluted to 1.00 mL with PBS (pH 7.4) buffer. The pre-samples (1.0 mL) were shaken evenly before incubated at 37 ${}^{\circ }$C for about 80 min. Then, fluorescence spectra were measured by luminescence spectrometer.

### 3.6. Interference Study

To evaluate the system selectivity toward glucose detection, 500 μmol solution of other common substances that commonly exist in the human body—Mg${}^{2+}$, Zn${}^{2+}$, Ca${}^{2+}$, Na${}^{+}$, K${}^{+}$, pepsin, BSA, GSH, and Cys — were prepared with 0.5 μg/mL HRP, 0.05 mM catechol, 0.05 mM GOx and 5.0 μmol/L GQDs in PBS buffer (pH 7.4), respectively.

### 3.7. Fluorescence Detection of Glucose in Human Blood Serum

To further test detection in real biological samples, two human blood samples, obtained by venipuncture from drug-free subjects, were used to assess the practicability of the GQDs-double enzyme system. After centrifugation at 10,000 rpm at room temperature for 10 min, the samples offering the best analytical performance for the assay were determined. The supernatant was stored under −20 ${}^{\circ }$C as a stock solution before the assay. The reaction serum samples were spiked with various concentrations of glucose and phenol–GQDs–double enzyme system before fluorescence detection.

For the determination of total glucose, human serum samples were prepared and diluted without any pre-treatment, except for a 100 times dilution. The fluorescence spectra for spiked serum samples and serum samples were recorded. By drawing the linear dependence relation of fluorescence intensity and the glucose level, quantification of glucose in the samples could be calculated. The measurement of two samples were repeated three times.

## 4. Conclusions

In conclusion, a simple and sensitive approach for glucose determination based upon the combination between specific enzymatic reaction and quenching effect of GQDs is reported. The reaction between the glucose and GQDs was accomplished by the addition of GOx, HRP and phenol. Glucose was oxidized by GOx; the formed H${}_{2}$O${}_{2}$ oxidized phenol via the catalysis by HRP. The oxidation product, quinone, caused the conspicuous quenching effect on GQDs due to efficient electron transfer from GQDs to quinone. The analysis results suggest the quenching degree was proportional to glucose concentration, which means the proposed approach could be successfully applied for the quantization of the glucose.

The bio-probe shows a high sensitivity ascribed to the ultrahigh optical properties of GQDs, and achieves high selectivity towards glucose attributable to the specificity and the unique affinity of the enzyme coupling method. The present approach is applicable for detecting glucose in human serum samples and have the potentiality to be extended to other systems. The present approach provided a pathway for the determination of the substance, which can generate glucose or H${}_{2}$O${}_{2}$. By combining fluorescent materials with the enzyme-coupled system, selective optical methods can have a more comprehensive application in future development. 

## Figures and Tables

**Table 1:** Comparison of different methods for the determination of glucose.

Method	System	Linear Range (μM)	LOD (μM)	Reference
Colorimetry	Graphene oxide/GOx/TMB	1–20	1	[49]
Colorimetry	Au–PtNCs/GOx/TMB	5–55	2.4	[50]
Electrochemistry	Graphene quantum dots/GOx	5–1270	1.73	[51]
Fluorometry	Hemin–functionalized GQDs/GOx	9–300	0.1	[42]
Fluorometry	Si quantum dots/GOx	5–650	0.68	[52]
Fluorometry	C–dots/AgNPs/GOx	2–100	1.39	[53]
Fluorometry	Hb–AuNCs/GOx	0.5–1000	1.65	[54]
Fluorometry	TA/Fe–Phen–CFs	0.5–200	0.19	[55]
Fluorometry	Graphene quantum dots/GOx/HRP/phenol	0.2–10	0.08	This paper

**Table 2:** Glucose determination in dilute serum samples (*n* = 3).

Samples	Added Glucose (μM)	Measured ( μM)	Recovery (%)	RSD (%)
Serum 1	2	1.97	98.5	3.94
Serum 2	5	5.11	102.0	2.44

## Abbreviations

GQDs	Graphene quantum dots
HRP	horseradish peroxidase
GOx	glucose oxidase
H${}_{2}$O${}_{2}$	hydrogen peroxide
